# Knowledge, risk perception, and condom utilization pattern among female sex workers in Dire Dawa, Eastern Ethiopia 2016: a cross-sectional study

**DOI:** 10.11604/pamj.2019.32.185.16574

**Published:** 2019-04-16

**Authors:** Hailemariam Mekonnen Workie, Teshager Worku Kassie, Tewodros Tesfa Hailegiyorgis

**Affiliations:** 1 Haramaya University, College of Health and Medical Sciences, School Of Nursing and Midwifery, Harar, Ethiopia; 2 Department of Medical Laboratory Science, College of Medical and Health Sciences, Haramaya University, Harar, Ethiopia

**Keywords:** HIV/AIDS, knowledge, risk perception, condom, utilization, female sex workers, Ethiopia

## Abstract

**Introduction:**

In 2015, in Dire Dawa administration city, adult HIV prevalence was 3.26 with 9,523 HIV positive population, & 251 annual AIDS deaths. Female sex workers are one of the high-risk groups for contracting HIV. Therefore, this study has assessed the level of HIV/AIDS knowledge, risk perception and condom utilization pattern among female sex workers' in Diredawa city.

**Methods:**

A cross-sectional study was conducted from April 15-June 25, 2016, in Dire Dawa among 156 female sex workers using convenient sampling method. Respondents were interviewed face-to-face using a pretested questionnaire. Training was provided to the data collectors and supervisors. Close supervision was done and double data entry was performed. Then the data were checked for completeness, consistency and entered into Epi Info v3.1 and analyzed using SPSS v20. The descriptive statistical analysis was used to compute frequency, mean, mode and proportion of the findings of this study. The results were presented using tables, charts, graphs, and texts.

**Results:**

Among the 156 female sex workers (FSWs), 99 (63.5%) had been working on commercial sex for more than one year, 92 (59%) were usually street-based, and 80 (51.3%) had partners between 2-3 per night. Only, 17 (10.9%) respondents mentioned three and above ways of HIV/AIDS transmission and prevention methods. Less than two-thirds (64.1%) of FSWs used a condom with all partners. One hundred thirty-eight (88.5%) of participants were engaged in unsafe sexual practice at least once since their engagement in sex work. Majority of FSWs (85.3%)) believed that their occupation is hazardous and 145 (92.9%) reported that they were unhappy being a commercial sex worker. Regarding risk perception, 79 (50.64%) and 37 (23.7%) of respondents perceived their chances of contracting HIV/STIs to be high and moderate respectively.

**Conclusion:**

Knowledge about HIV/STIs and magnitude of condom utilization were good. However, a high number of unsafe sex and unsatisfactory risk perception attitudes were observed. Thus, a collaborative effort is needed to create awareness regarding risk perception attitude and increase the level of their practice towards the prevention of unsafe sex.

## Introduction

Half of the world’s population suffers from infections including HIV/AIDS, hunger and unsustainable access to safe water and basic sanitation [1]. HIV/AIDS continues to be a major global public health problem, having claimed more than 35 million lives so far. At the end of 2017, there were approximately 36.9 million people living with HIV with 1.8 million people becoming newly infected and 940 000 people who died from HIV-related causes globally [2, 3]. Likewise, in 2015, there were 36.7 million people living with HIV, and 1.1 million deaths [4, 5]. The African region accounts for over 2/3rd of the global total of new HIV infections [2]. Sub-Saharan Africa has been the most seriously HIV stricken region, accounting for seventy-one percent of all new infections in adults and children globally [2, 6, 7]. Ethiopia is one of the country in the world that has the largest HIV/AIDS burdens and experiencing a ‘generalized’ HIV epidemic (epidemic defined as the HIV seroprevalence level amongst sexually active adults in the general population is greater than one percent) with an estimated 741,478 people living with HIV and 16,865 AIDS-related death in 2015 [8]. In the same year, in Dire Dawa administration city, adult (15+ years) HIV prevalence was 3.26 with 120 new HIV infection, 9,523 HIV positive population, 251 annual AIDS deaths and 3,690 total AIDS orphan [8]. Sex workers and their clients, men who have sex with men, people who inject drugs, people in prisons and other closed settings, and transgender people are key populations that are at increased risk of HIV irrespective of epidemic type or local context. This key populations often have legal and social issues related to their behavior that increase vulnerability to HIV and reduce access to testing and treatment programmes. In 2017, an estimated 47% of new infections occurred among key populations and their partners [2].

Furthermore, it is recognized that HIV seroprevalence is considerably above this level amongst female sex workers and mobile populations [9]. Female sex workers are the highly vulnerable groups that are at high risk of contributing to sexually transmitted diseases [10, 11]. Even though street-based female sex workers (FSWs) are highly vulnerable to HIV, only about one in every three of them receive adequate HIV prevention services and medical care [12, 13]. Female sex workers encountered numerous challenges from their clients that expose them to unsafe sex, because of their hazardous occupational situation [14-16]. Therefore, effective, consistent and correct utilization of condom is one of the best preventive intervention methods of HIV and others STIs targeted toward female sex workers. It would avert two-thirds of an incident of HIV infections [17-20]. Use of male and female condoms, increasing the availability, accessibility, and affordability of condom among female sex workers are an essential component to reduce the enormous consequences and costs of STIs and unintended pregnancies [1, 19]. Regardless of many efforts in the world, there is still no vaccine & cure for HIV/AIDS [2, 4]. However, effective antiretroviral drugs can control the virus & help prevent transmission so that people with HIV, & those at substantial risk, can enjoy healthy, long & productive lives [2]. Despite these, there is still a tangible and visible gap between the desired goal to achieve and the present level of condom utilization among FSWs [12, 21]. In addition, unsatisfactory and inadequate HIV and condom utilization knowledge and negative risk perception attitude are the challenges among female sex workers to fight HIV/STIs [22, 23]. Thus, this study assessed the level of female sex workers' HIV/AIDS knowledge, risk perception and condom utilization pattern in Dechatu, Dire Dawa, Eastern Ethiopia.

## Methods

**Study period and area:** This study was conducted in Dire Dawa city from April 15-June 25, 2016. Dire Dawa is found 515km away from the capital city of Ethiopia, and 315km to Djibouti. The Population size was about 398,429 with 49% male and 51% female based on the 2007 Ethiopian census. The city has 9 urban and 38 rural Kebele with 54,575 urban and 22,240 rural households. Dechatu is one of kebele among the 9 urban kebele with the total population size of 22,205. It has 13 ketena that is bounded by Coneal in the east, Kezira from the North, Addisketma from the South and Legahara from the West. It has one public school, one private school, and one public health center.

**Study design and participants:** A cross-sectional study was conducted in Dire Dawa to assess female sex workers HIV/AIDS knowledge, risk perception, and condom utilization pattern. All female sex workers between the ages of 15-49 years living in the study area for at least 6 months were included for an interview but those who had a physical impairment (unable to hear and speak) and mentally ill were excluded from the study.

**Sample size determination and technique:** the sample size (n) required for the study was determined using a single population proportion formula

$$(n={\left(za/2\right)}^{2}\;p(1-p/d^{2}));$$

whereas n = the required sample size, Za/2(1.96): significance level at α=0.05 with 95% confidence interval, p: proportion of condom utilization among female sex workers (89.5%) [24], d: margin of error (5%) and 10% non-response rate. Therefore, the formula would provide the sample size of n=156. Convenient sampling method was used to recruit study participants as it was not possible to have a sampling frame for this population study, and their hidden and hard-to-reach behavior.

**Measurement and data collection procedure:** Data was collected face to face using a pretested questionnaire that was adapted from different kinds of literature [25-27] and modified to suit the study objectives. It contains female sex workers' socio-demographic status, HIV/AIDS and condom knowledge, risk perception and condom utilization pattern. Three BSc nurses and 1 MSc nurse were selected for data collection and supervision respectively.

**Data quality control:** The data quality was assured by using different methods. The standard and structured questionnaire was used. The questionnaire was prepared in English and translated into the local language (Amharic) for data collection and then retranslated back into English for analysis. Two days of training was provided to the data collectors and supervisors on the data collection tool and the data collection procedures. Then the questionnaire was pretested on 5% of the sample size to ensure its validity. Findings from the pretesting were utilized for modifying and adjusting of the instrument and interviewing technique. Data collectors were supervised closely by the supervisors and the principal investigators. Completeness of each questionnaire was checked by the principal investigator and the supervisors on daylily basis. Double data entry was done by two data clerks and the consistency of the entered data was cross-checked by comparing the two separately entered data on Epi Info.

**Data processing and analysis:** After collection of the data, each questionnaire was thoroughly reviewed for completeness and consistency by the data collectors, supervisor and investigators. Then the data were entered into Epi Info version 3.1 and analyzed by using SPSS for window version 20. Descriptive statistics was employed to examine the findings using frequency, mean, mode and proportion. The results were presented using tables, charts, graphs, and result statements.

**Ethics approval and consent to participate:** The research proposal was ethically cleared by the Department Research and Ethical Review Committee (DREC) and approved by the school of nursing & midwifery, college of health and medical science, Haramaya University. Informed verbal consent, which was approved by ethics committee, was obtained from each study subject prior to the interview after the purpose of the study was explained to them. If the respondents were under 16, consent to participate was taken from the parental/legal guardian. Confidentiality of the information was assured and privacy of the respondent was maintained.

## Results

A total of number of 156 female sex workers were involved in the data collection with a 100% response rate. Therefore, 156 respondents' data were included in the analysis.

**Sociodemographic characteristics of the respondents:** Half of the respondents (50%) were between the age group of 20-24 years old and 15-19 years (44.9%) with a mean age of 19.5 and the median age of 20. The mean and median age of the participants at first sex and first selling sex were 17 and 18 years and 20 and 19 years old respectively. Majority of the female sex workers (88.5%) were ever unmarried. Concerning the educational status of the respondents, less than half (44.2%) of the FSWs had attended from 5 to 8 grade, and nearly one-quarter of them (24.4%) were illiterate but none of the respondents attained higher education. Regarding, residence and employment, all of the FSWs reported that they were currently living in Dire Dawa town. Only 12% of them were involved in income generating activities other than sex work. From the total respondents, 51.3% were orthodox religion followers and the remaining were Muslim. Seventy-six (48.7%) of FSWs were from Amhara ethnicity (Table 1).

**Table 1:** Sociodemographic characteristics of FSWs in Dechatu, Diredawa, eastern Ethiopia 2016 (n=156)

Variables		Frequency	Percent
Age	15-19 years	70	44.9
	20-24 years	78	50
	25-29 years	6	3.8
	>=30 years	2	1.3
	Total	156	100.0
Marital status	Ever married	18	11.5
	Ever unmarried	138	88.5
	Total	156	100.0
Religion	Orthodox	80	51.3
	Muslim	76	48.7
	Others	0	0
	Total	156	100.0
Ethnicity	Oromo	66	42.3
	Amhara	76	48.7
	Somali	4	2.6
	Other	10	6.4
	Total	156	100.0
Educational status	Illiterate	38	24.4
	1-4	44	28.2
	5-8	69	44.2
	9-12	5	3.2
	12+	0	0
	Total	156	100.0

**Figure 1:**
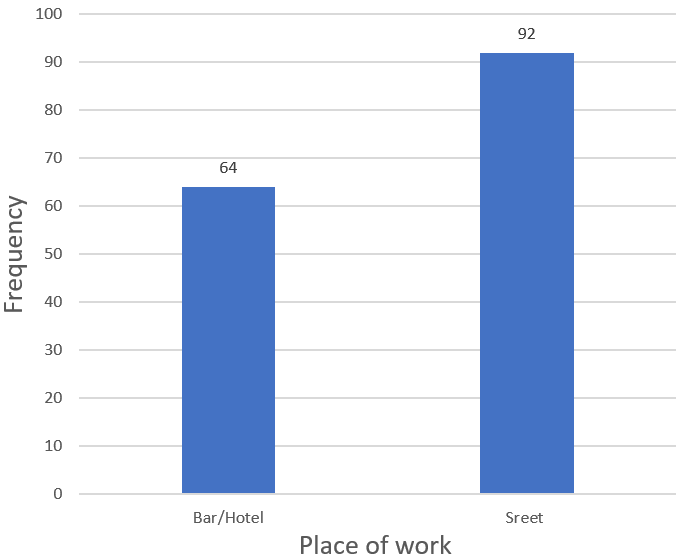
Usual place of female sex workers to pick up their clients in Dechatu, Diredawa, eastern Ethiopia 2016 (n=156)

**Usual place of respondents to pick up their clients:** In relation to the place of female sex workers to pick their clients, 92 (59%) were usually street-based and the remaining 64 (41%) were bar/hotel based to get their partner for a single time or more than single moment (Figure 1).

**Female sex workers (FSWs) engagement period, number of partners, average income:** Among the involved FSWs for an interview, 99 (63.5%) reported that they had been working on commercial sex for more than a year and 21 (13.5%) had been engaging in commercial sex for less than six months. We also asked them about their average number of partners per night. Accordingly, 80 (51.3%), 48 (30.8%) and 28 (18.0%) of them had between two and three partners, more than three partners and one partner per night respectively. Among those who had reported having more than three partners per night, 42 (87.5%) were street-based FSWs and the remaining 6 (12.5%) were bar/hotel based. Concerning their average income per night, 78 (50%) of FSWs had reported that they usually got fifty-one to eighty Ethiopian Birr per night (Table 2).

**Table 2:** FWS’s engagement period, number of partners, average monthly income in Dire Dawa town, 2016 (n=156)

Variables			Frequency	Percent
Engagement period to Commercial sex actives	More than one year	Yes	99	63.5
		No	57	36.5
	Six months to one year	Yes	36	23.1
		No	120	76.9
	Less than six months	Yes	21	13.5
		No	135	86.5
Number of partner per night	<=1	Yes	28	18.0
		No	128	82.0
	2-3	Yes	80	51.3
		No	76	48.7
	>3	Yes	48	30.8
		No	108	69.2
Average income per night	<20 Ethiopian Birr(ETB)	Yes	2	1.3
		No	154	98.7
	20-50 ETB	Yes	23	14.7
		No	133	85.3
	51-80 ETB	Yes	78	50.0
		No	78	50.0
	>80 ETB	Yes	53	34.0
		No	103	66.0

**Female sex workers Knowledge about HIV/AIDS and Condom:** Regarding HIV/AIDS transmission and prevention methods, 149 (95.5%) of FSWs mentioned one method, 115 (73.7%) mentioned two methods and the remaining 17 (10.9%) mentioned three and above diffent methods of HIV/AIDS transmission and prevention. (Table 3). Female sex workers knowledge of condom was measured by asking about the ability of the condom to prevent HIV/AIDS and its availability, accessibility & affordability. Based on these, 138 (88.5%) of FSWs believed that condom can prevent HIV/AIDS. All of the respondents reported that condom is available in nearby their occupational area (such as shops, pharmacy, health facilities, and bars) but only 16.7% of them Obtained condom freely as they want and 75% of females obtained in reasonable cost (purchasing in the exchange of less than one Ethiopian birr per pack of condom) (Table 3).

**Table 3:** Knowledge of FSWs about HIV/AIDS transmission and prevention methods, Dechatu, Diredawa, eastern Ethiopia, 2016 (n=156)

Variables			Frequency	Percentage
Did you know about HIV transmission and prevention methods?	Yes		154	98.7
	No		2	1.3
	Total		156	100.0
Mention about HIV transmission and prevention methods	One		149	95.5
	Two		115	73.7
	Three and above		17	10.9
Do you think that condom is	Accessible	Yes	154	98.7
		No	2	1.3
	Available	Yes	156	100
		No	0	0
	At a reasonable cost	Yes	117	75
		No	39	25
	Obtained freely	Yes	26	16.7
		No	130	83.3
	prevent HIV transmission	Yes	138	88.5
		No	18	11.5

**Figure 2:**
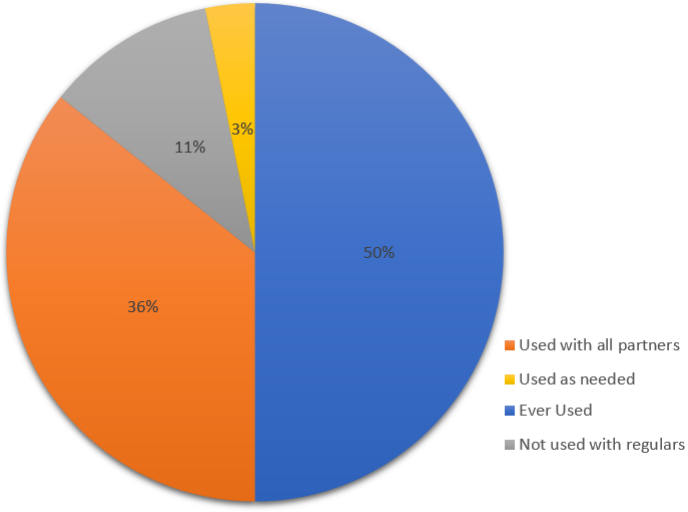
Condom utilization pattern among female sex workers in Dechatu, Diredawa, eastern Ethiopia 2016 (n=156)

**Condom utilization pattern and associated sexual behaviors:** Regarding condom utilization, 140 (89.7%) of FSWs always used condoms, 100 (64.1%) used condoms with all partners, 9 (5.8%) used condoms as needed and 31 (19.9%) of them didn't use condoms with their regular partners during sexual intercourse (Figure 2). In contrast, one hundred and thirty-eight (88.5%) of the females, had been engaged in unsafe sexual practice at least once since their engagement in sex work. The main reason listed by them to engage in unsafe sex was due to deliberate acts of their partners (34.1%) like: lack of condom utilization skill by their partners (26.1%), paid beyond normal (12.3%), forced by their client (08.0%), alcohol intoxication (04.3%) and the rest didn't remember the reasons. Respondents’ ability to convince their partners about unsafe sex was also unsuccessful. From those who were asked for unsafe sex by their partner at least once since their engagement of sex work, only 20% of them tried to convince their partner/client but the remaining (80%) couldn't. As a result, they quarreled and disagreed (Table 4). As like other variables, consistency in utilization of condom was also assessed. Among 140 (89.7%) FSWs who reported that they had used a condom, 100 (71.4%) of them used with all partners, 31 (22.1%) with their regular partners (‘lovers’) and 9 (6.4%) said that they had used as needed (Table 5).

**Table 4:** Unsafe sexual practices and underline causes among female sex workers of Dechatu Diredawa, Ethiopia, 2016 (n=156)

Variables		Frequency	Percent
Did you ask for unsafe sex by your client at least once since your engagement	Yes	125	80.1
	No	31	19.9
	Total	156	100.0
Have you tried to convince your partner/client regarding unsafe sex	Yes	25	20.0
	No	100	80.0
	Total	125	100.0
Have you ever practiced unsafe sex at least once since your engagement?	Yes	138	88.5
	No	18	11.5
	Total	156	100.0
What was your reason to engage in unsafe sexual practice	Paid beyond normal	17	12.3
	Forced	11	08.0
	Alcohol intoxication	6	04.3
	Deliberate action of partners	47	34.1
	Lack of condom utilization skill by their partners	36	26.1
	Not known	21	15.2
	Total	138	100.0

**Table 5:** Consistency utilization of condom among FSWs of Dechatu, Diredawa, Ethiopia, 2016 (n=156)

Variable			Frequency	Percent
Have you used a condom during sexual intercourse with your partner	Yes		140	89.7
	No		16	10.3
With who do you use a condom?	With all partners	Yes	100	71.4
		No	56	28.6
		Total	140	100.0
	With regular partners	Yes	31	22.1
		No	109	77.9
		Total	140	100.0
	Use as needed with anyone	Yes	9	6.4
		No	131	93.6
		Total	140	100.0

**Risk perception of female sex workers towards HIV and/or other Sexual transmitted infections:** FSWs were asked about the hazards of commercial sex from the angles of its potency in exposing themselves to HIV/STIs. One hundred thirty-three (85.3%) of them stated that this occupation was hazardous, 15 (9.6%) hadn't had that much hazard and the remaining 8 (5.1%) said the occupation hadn't had any risk of exposing them to HIV/STIs. About the level of job satisfaction, 145 (92.9%) described that they were unhappy with the commercial sex, 5 (3.20%) reported that they were happy and the rest 6 (3.84%) said that they didn't know. The reason forwarded by those who had said ‘unhappy’ for their engagement in commercial sex was family conflict [84 (53.8%)], those who couldn't get another job were [38 (37.17%)], those who faced peer pressure were [10 (6.41%)] and those who did not know were [4 (2.56%)].

FSWs were also requested to rank their chances of becoming infected with HIV/STIs based on their past sexual and other risky behaviors. Consequently, 79 (50.64%), 37 (23.7%), 25 (16%), and 15 (9.61%) of respondents perceived their chances of contracting HIV to be high, moderate, low/nil respectively, and the remaining couldn't rank their chances contracting HIV/STIs infection. Respondents were also asked to give reasons for their personal perceived risk of HIV/STIs infection. The majority [65 (42%)] of FSWs who perceived their risk as high or moderate, said that they had encountered high failures with condoms during sexual practice and 36 (23%) said that they were forced to practice unsafe sex by persons whom they suspect for having HIV/STIs. On the other hand, those FSWs who perceived their risk of HIV infection as low or nil gave a common reason of being very condom concious [13 (8%)], had had only one partner with whom they practice unsafe sex with [7 (4.5%)] and the remaining [5 (3%)] said that they hadn't practiced sexual intercourse with a client whom they suspect or know has HIV.

## Discussion

The finding of this research revealed that most of the respondents (95.5%), knew at least one method of HIV/STIs transmission and prevention. This finding is significantly higher than a cross-sectional study conducted in Northwest Ethiopia [28]. The possible reason for the difference might be due to the expansion of media. Following these, there will be an increased awareness among female commercial sex workers about the transmission and prevention methods of HIV/STIs. In this study, 138 (88.5%) of FSWs believed that condom can prevent HIV/AIDS. This is in line with a study conducted in Arsi, Addis Ababa and Debre Berhan-Ethiopia (86%) [29] and Northwest Ethiopia ( 80.1%) [28]. The similarity of this finding could be due to the resemblance of socio-culture, the way of living of the FSWs and the nature of the work.

Condom utilization among female sex workers in this study (89.7%) is comparable with findings in Northwest Ethiopia (84.2%) [28] and Phnom Penh, Cambodia (86.9%) [30] but much higher than the studies conducted in Arsi, Addis Ababa and Debre Berhan-Ethiopia (33%) [29], Abidjan, Côte d´Ivoire (11%) [31], and Pretoria, South Africa (43%) [32]. The high promotion of condom use in the country and its access freely or at an affordable price may perhaps be the possible reasons for the differences. From the total, 138 (88.5%) of female sex workers were engaged in unsafe sexual practice at least once since their engagement in sex work. On the other hand, a study conducted in Metemma Yohannes, Northwest Ethiopia showed that [303 (63.9 %)] of FSWs engaged in unsafe sex [33]. The main reason to engage in unsafe sex might be due to deliberate acts of their partners, lack of condom utilization skill by their partners, paid beyond expected, forced by their client, and alcohol intoxication. From 140 (89.7%) of FSWs who reported that they had used a condom, 100 (71.4%) of them used a condom with all partners. This finding is lower than studies conducted in Savannakhet, Lao PDR (97%), Southern India, (85%), and Mekelle, Ethiopia (94.2%) [34-36]. On the other hand, it is higher than studies conducted in Northwest Ethiopia (47.7%), Arsi, Addis Ababa and Debre Berhan-Ethiopia (59%), Zhejiang province, China (50.5%), and Democratic Republic of Congo (40%) [28, 29, 37, 38]. The discrepancy might be due to the difference in geographical area, sample size, socio-economic and culture. FSWs were also asked about their average number of partners per night. Accordingly, 80 (51.3%) of the females had between two and three partners per night respectively. Unlike this, 64 (16.5%) FSWs had two or more sexual clients per day in Fenoteselam, Ethiopia [38]. The difference could be due to that the respondents in this study are living in a more urbanized and civilized city of the country compared to those living in Fenoteselam, Ethiopia.

In this study, 133 (85.3%) of FSWs believed that this occupation was hazardous and had a risk of exposing them to HIV, 79 (50.64%) and 37 (23.7%) of respondents perceived their chances of contracting HIV to be high and moderate respectively. On the opposite, 67%, 68% and 58% of FSWs in Addis Ababa, Debre Berhan Arsi-Hitosa-Ethiopia didn't feel vulnerable to HIV/AIDS respectively [29]. The educational background is the probable reasons for these difference. Regarding the level of job satisfaction, 145 (92.9%) reported that they were unhappy with the commercial sex and only 5 (3.20%) reported that they were happy. A cross-sectional survey conducted in Queensland, Australia among 247 female sex workers showed that most sex workers reported positive job satisfaction. The discrepancy may due to the payment difference and the care given by their sexual partner.

## Conclusion

Knowledge about HIV/STIs and magnitude of condom utilization were good. However, a great number of female sex workers had engaged in unsafe sex and only half of them perceived that they have the probability of a high chance of contracting HIV/STIs. Thus, a collaborative effort is needed to create awareness among FSWs regarding risk perception attitude and increase the level of their practice towards the prevention of HIV/STIs and unsafe sex.

### What is known about this topic

- Female sex workers are high-risk groups for the contracting and transmiting of STIs/HIV;
- Female sex workers encountered numerous challenges from their clients that expose them to unsafe sex, because of their hazardous occupational situation;
- Effective, consistent and correct utilization of condom would avert two-thirds of an incident of HIV infections.

### What this study adds

- Most female sex workers in the study setting mentioned condom as preventive method for STIs/HIV;
- An encouraging condom utilization was observed among female sex workers in the last six months during sexual intercourse with their partners;
- Majority of female sex workers have been engaged in unsafe sexual practice at least once since their engagement in sex work, mainly due to deliberate acts and lack of condom utilization skill by their partners.

## Competing interests

The authors declare no competing interests.
